# Rarefaction, Alpha Diversity, and Statistics

**DOI:** 10.3389/fmicb.2019.02407

**Published:** 2019-10-23

**Authors:** Amy D. Willis

**Affiliations:** Department of Biostatistics, University of Washington, Seattle, WA, United States

**Keywords:** bioinformatics, computational biology, ecological data analysis, latent variable model, reproducibility, measurement error

## Abstract

Understanding the drivers of diversity is a fundamental question in ecology. Extensive literature discusses different methods for describing diversity and documenting its effects on ecosystem health and function. However, it is widely believed that diversity depends on the intensity of sampling. I discuss a statistical perspective on diversity, framing the diversity of an environment as an unknown parameter, and discussing the bias and variance of plug-in and rarefied estimates. I describe the state of the statistical literature for addressing these problems, focusing on the analysis of microbial diversity. I argue that latent variable models can address issues with variance, but bias corrections need to be utilized as well. I encourage ecologists to use estimates of diversity that account for unobserved species, and to use measurement error models to compare diversity across ecosystems.

## 1. Introduction

Alpha diversity metrics summarize the structure of an ecological community with respect to its richness (number of taxonomic groups), evenness (distribution of abundances of the groups), or both. Because many perturbations to a community affect the alpha diversity of a community, summarizing and comparing community structure via alpha diversity is a ubiquitous approach to analyzing community surveys. In microbial ecology, analyzing the alpha diversity of amplicon sequencing data is a common first approach to assessing differences between environments.

Unfortunately, determining how to meaningfully estimate and compare alpha diversity is not trivial. To illustrate, consider the following example where the alpha diversity metric of interest is strain-level richness of a microbial community (the total number of strain variants present in the environment). Suppose I conduct an experiment in which I take a sample from Environment A and count the number of different microbial taxa present in my sample. I then take a sample from Environment B, count the number of different taxa in that sample, and compare it to the number of taxa in Environment A. I am likely to observe higher numbers of different taxa in the sample with more microbial reads. The library sizes can dominate the biology in determining the result of the diversity analysis (Lande, 1996).

Rarefaction is a method that adjusts for differences in library sizes across samples to aid comparisons of alpha diversity. First proposed by Sanders (1968), rarefaction involves selecting a specified number of samples that is equal to or less than the number of samples in the smallest sample, and then randomly discarding reads from larger samples until the number of remaining samples is equal to this threshold (see Hurlbert, 1971 for a deterministic version). Based on these subsamples of equal size, diversity metrics can be calculated that can contrast ecosystems “fairly,” independent of differences in sample sizes (Weiss et al., 2017).

Unfortunately, rarefaction is neither justifiable nor necessary, a view framed statistically by McMurdie and Holmes (2014) in the context of comparison of relative abundances. In this article, I discuss why unequal sample sizes appear to cause special problems in the analysis of alpha diversity. I introduce a statistical perspective on the estimation of alpha diversity, and argue that a common view of diversity indices is causing fundamental issues in comparing samples. Without advocating for any particular model of microbial sampling, I suggest a general approach to comparing microbial diversity, one which accounts for uncertainty in estimating diversity metrics. However, since estimates for alpha diversity metrics are heavily biased when taxa are unobserved, comparing alpha diversity using either raw or rarefied data should not be undertaken. I describe statistical methodology for alpha diversity analysis that adjusts for missing taxa, which should be used in place of existing common approaches to diversity analysis in ecology. While the focus of the examples is microbiome data analysis, the issues and discussion are equally applicable to macroecological data analysis. Furthermore, this discussion applies equally to diversity analyses performed at the strain, species, or other taxonomic level.

## 2. Measurement Error and Variance in Microbiome Studies

Imagine that we had complete knowledge of every microbe in existence, including identity, abundance and location. To compare microbial diversity, we would define specific environments (e.g., the distal gut of women aged 35 living in the contiguous U.S.) and compare diversity metrics across different ecological gradients (e.g., with or without irritable bowel syndrome diagnoses). Alpha diversity could be compared exactly, because we would know entire microbial populations with perfect precision.

Unfortunately, we do not have knowledge of every microbe. We take samples from environments, and investigate the microbial community present in the sample. We use our findings about the sample to draw inferences about the environment that we are truly interested in. The samples are not of particular interest, except that they reflect the environment from which they were sampled. As we sample more and more of the environment using larger samples, we get closer to understanding the true and total microbial community of interest. This means that as we increase sampling, our calculation of any diversity metric [e.g., richness (Fisher et al., 1943), Shannon index (Shannon, 1948), and Simpson index (Simpson, 1949)] approaches the value of that diversity metric as calculated using the entire population.

Observing small samples from a large population is not an experimental set-up unique to microbial ecology: it is almost universal in statistics. The set-up where an estimate of a quantity converges to the correct value as more samples are obtained is also well understood in statistics. The unique property of microbiome experiments and alpha diversity analysis is that samples do not faithfully represent the entire microbial community under study. There is unadjusted error in using our samples as proxies for the entire community.

To illustrate this distinction, I contrast microbial diversity experiments with a non-microbial experiment. Suppose we are interested in modeling the CO_2_ flux of soil treated with different amendments. We would measure the flux of equally sized soil sites treated with the different amendments, performing biological replicates using multiple sites for each amendment. To assess if the amendments affect the flux, we would fit a regression-type model (such as ANOVA) to flux with amendment as an explanatory variable. Implicitly, this model acknowledges that we can assess the flux with high precision; that is, the margin for error for determining flux is negligible.

Now suppose we knew that our flux-measuring machine consistently underestimated flux by exactly 5 units. We would adjust for the measurement error by adding 5 units to each measurement before comparing them. But what happens when we have random measurement error? If the measurement error on the machine was random (e.g., with 0 mean and variance of 1 unit for all amendments), this would not affect any particular amendment. However, detecting a difference between the effects of amendment on flux would be more challenging statistically: we would require more samples to detect a true difference compared to the case without measurement error. To account for the additional experimental noise, we would use a model that would account for measurement error in assessing differences between amendments. If the variance in the measurement error was 1 unit for amendment A but 5 units for amendment B, we would similarly adjust with a measurement error model.

To decide if measurement error must be accounted for when observations are made in an experiment, it is necessary to consider the effect of repeating the observational process on the same experimental unit. In the flux experiment, this would involve measuring the flux of the same soil sites again using the same experimental conditions. Without measurement error in the observations, we would consistently observe the same flux measurement, while if we had random measurement error, we would most likely observe slightly different flux measurements. Because technical replicates in microbiome experiments yield different numbers of reads, different community compositions, and different levels of alpha diversity, we have measurement error in microbial experiments. We currently do not account for measurement error in microbial diversity studies.

## 3. Bias in Estimating and Comparing Alpha Diversity

While measurement error in microbiome studies affects all analyses of microbiome data, alpha diversity is particularly affected because commonly used estimates of alpha diversity are heavily biased compared to other estimation problems in microbial ecology (such as estimating relative abundances). Some tools to address problems with bias in alpha diversity exist in the statistical literature (Chao and Bunge, 2002; Willis and Bunge, 2015; Arbel et al., 2016; Willis and Martin, 2018). However, there are two incorrect practices surrounding alpha diversity that are preventing the uptake of statistically-motivated methodologies. The first practice is using biased estimates of alpha diversity indices. The second practice is treating alpha diversity estimates as precisely observed quantities that do not have measurement error.

**Figure 1:**
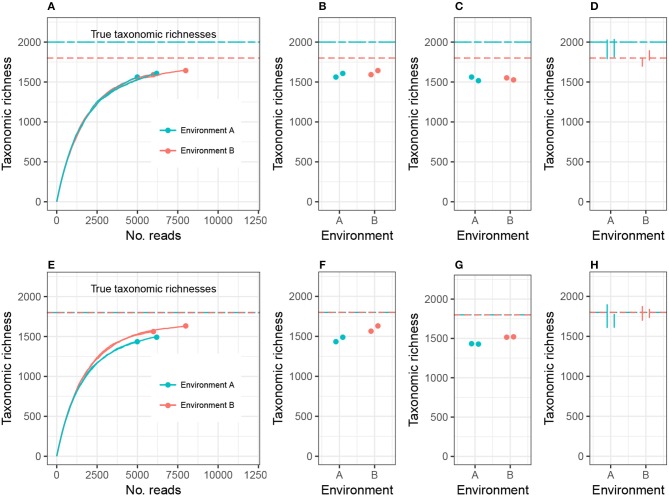
Expected sample taxonomic richness increases with number of reads **(A,E)**. Comparing sample taxonomic richness can therefore often lead to incorrect conclusions about true richness **(B,F)**. Rarefying samples to the same number of reads can also lead to incorrect conclusions **(C,G)**. Adjusting for unobserved taxa and accounting for uncertainty in the estimate correctly detects both true **(D)** and false **(H)** differences in richness. While the example employed here concerns microbial richness, the same argument applies to macroecological richness, as well as other alpha diversity indices.

To clarify this discussion, I will focus on taxonomic richness (the simplest case), and later generalize the argument to other alpha diversity metrics. Consider the setting in Figure 1A, where we are investigating 2 different environments, and Environment A's richness (call it *C*_*A*_) is higher than Environment B's richness (*C*_*B*_). Suppose we have two biological replicates of samples from each environment: *n*_*A*1_ and *n*_*A*2_ reads from Environment A, *n*_*B*1_ and *n*_*B*2_ reads from Environment B, and *n*_*A*1_ < *n*_*B*1_ < *n*_*A*2_ < *n*_*B*2_. Let *c*_*ij*_ be the observed richness of environment *i* on replicate *j*. As may commonly occur in practice, *c*_*A*1_ < *c*_*A*2_ < *c*_*B*1_ < *c*_*B*2_.

There are currently two commonly used methods for comparing alpha diversity. The first method, Figure 1B, is to use the estimates *c*_*A*1_, *c*_*A*2_, *c*_*B*1_, and *c*_*B*2_, and perform modeling and hypothesis testing (such as ANOVA) as if both the bias and variance of these estimates were zero (see, for example, Makipaa et al., 2017). In the setting of Figure 1A, this leads to the erroneous conclusion that Environment A has lower richness than Environment B. The second method is to generate a normalized, or rarefied sample by randomly discarding reads from all samples until each sample has *n*_*A*1_ reads (the number of reads in the smallest sample), Figure 1C. The resulting rarefied richness levels are then *c*_*A*1_, $c_{A2}^{\prime }$, $c_{B1}^{\prime }$, and $c_{B2}^{\prime }$. These estimates are then used for modeling and hypothesis testing (see, for example, Arora et al., 2017). This leads to the conclusion that Environment A and Environment B do not have significantly different richnesses, and the estimates of richness are far below the actual richnesses of each ecosystem (there is substantial negative bias in the estimates), prohibiting comparison of richness across different experiments. Furthermore, not all information collected from the samples was used in making the comparison.

Here I advocate for a third strategy: adjust the sample richness of each ecosystem by adding to it an estimate of the number of unobserved species, estimate the variance in the total richness estimate, and compare the diversities relative to these errors (Figure 1D). This option has the advantages of leveraging all observed reads, comparing estimates of the actual parameter of interest (taxonomic richness), and accounting for experimental noise. In the case where the environments have equal richness (Figures 1E–H), this approach correctly detects equal richness, even when the abundance structures differ.

Modeling parameters observed with estimation error is not a new suggestion: this approach is from the field of statistical *meta-analysis*, where the results of multiple studies estimating the same effect size is compared (Demidenko, 2004; Willis et al., 2016; Washburne et al., 2018). In meta-analyses, larger studies need to be given more weight in determining the overall effect size, and this is incorporated into a meta-analysis via the smaller standard errors on the effect size estimates. Similarly, when comparing the response of different treatment groups in clinical trials, the number of subjects in each treatment group is accounted for in a comparison of the overall treatment effect. Adjusting for sample size when comparing different groups of observations without discarding data is widely prevalent in the sciences, and discarding data to adjust for unequal sample sizes is the exception. The strategy outlined here for modeling richness after adjusting for missing species adjusts for both bias and variance, thus accounting for library size differences and incomplete microbial surveys.

While the example discussed here is richness, this approach to estimating and comparing alpha diversity using a bias correction (incorporating unobserved taxa) and a variance adjustment (measurement error model) could apply to any alpha diversity metric. However, richness estimation has a well-studied statistical literature, and richness estimators that are adapted to microbiome data exist (see Bunge et al., 2014 for a review). The same is not true for other alpha diversity metrics. For example, the Chao-Bunge (Chao and Bunge, 2002) and breakaway (Willis and Bunge, 2015) estimators of taxonomic richness provide variance estimates, account for unobserved taxa, and are not overly sensitive to the singleton count (the number of species observed once). In contrast, the coverage adjusted entropy estimator of the Shannon index (Chao and Shen, 2003) provides variance estimates and accounts for unobserved taxa, but is extremely sensitive to the singleton count, which is often difficult to determine in microbiome studies. While alpha diversity estimation for microbiomes is an active area of research in statistics (Arbel et al., 2016; Zhang and Grabchak, 2016; Willis and Martin, 2018), there remain many features of microbial ecosystems (such as crosstalk between samples and spatial organization of microbes) that are not yet incorporated into statistical methodology for alpha diversity estimation. Despite this, alpha diversity estimates that account for unobserved taxa and provide variance estimates are vastly preferable to both plug-in and rarefied estimates, which do not account for unobserved taxa nor provide variance estimates.

## 4. Discussion

Plug-in estimates of many alpha diversity indices (including richness and Shannon diversity) are negatively biased for the environment's alpha diversity parameter, that is, they underestimate the true alpha diversity (Lande, 1996). Attempting to address this problem using rarefaction actually induces more bias. This is sometimes justified by claiming that rarefied estimates are equally biased. However, this is not generally true, because environments can be identical with respect to one alpha diversity metric, but the different abundance structures will induce different biases when rarefied. For example, Figure 1E shows two environments with different abundance structures but equal richness; rarefying gives the false impression of unequal richness (see also Lande et al., 2000). In this way, both sample richness and rarefied richness are driven by artifacts of the experiment (library size), and not purely the microbial community structure. In order to draw meaningful conclusions about the entire microbial community, it is necessary to adjust for inexhaustive sampling using statistically-motivated parameter estimates for alpha diversity. In order to draw meaningful conclusions regarding comparisons of microbial communities, it is necessary to use measurement error models to adjust for the uncertainty in the estimation of alpha diversity.

It has recently been argued that studying microbial diversity without context is distracting us from gaining insight into ecological mechanisms (Shade, 2016). To this criticism, I add misapplying statistical tools is undermining many analyses of alpha diversity. I encourage microbial ecologists to use estimates of alpha diversity that account for unobserved species, and to use the variance of the estimates in measurement error models to compare diversity across ecosystems.

## Author Contributions

AW wrote the manuscript and performed the data analysis.

### Conflict of Interest

The author declares that the research was conducted in the absence of any commercial or financial relationships that could be construed as a potential conflict of interest.
